# Role of erythritol in coronary heart disease, ischemic stroke, and venous thromboembolism: A Mendelian randomization analysis

**DOI:** 10.1097/MD.0000000000045187

**Published:** 2025-10-24

**Authors:** Yao Sun, Dingxin Sun, Jinqiao Wu, Ze Peng, Juan Jin

**Affiliations:** a The First Affiliated Hospital of Heilongjiang University of Chinese Medicine, Harbin, China; b Department of General Surgery, Shuangliao Hospital of Traditional Chinese Medicine, Shuangliao, China.

**Keywords:** coronary heart disease, erythritol, ischemic stroke, Mendelian randomization, venous thromboembolism

## Abstract

Erythritol, a widely used nonnutritive sweetener, has been linked to increased cardiovascular risk in observational studies. However, whether erythritol causally contributes to arterial and venous thrombotic diseases remains unclear. We performed a 2-sample Mendelian randomization (MR) study using 60 independent single nucleotide polymorphisms strongly associated with erythritol from a genome-wide association study of 8167 European individuals. Summary statistics for coronary heart disease, ischemic stroke, venous thromboembolism (VTE), pulmonary embolism (PE), and deep vein thrombosis (DVT) were obtained from the FinnGen consortium. The inverse-variance weighted method was the primary analysis, supplemented by MR-Egger, weighted median, and mode-based sensitivity analyses. Pleiotropy and heterogeneity were assessed. Genetically predicted higher erythritol levels were significantly associated with increased risks of coronary heart disease (odds ratio [OR] = 1.077, 95% confidence interval [CI]: 1.060–1.090) and ischemic stroke (OR = 1.157, 95% CI: 1.135–1.179), with consistent findings across sensitivity analyses and no evidence of pleiotropy. A suggestive association was observed for DVT (OR = 1.117, 95% CI: 1.077–1.158); however, causal effect directions for VTE and PE were inconsistent across MR methods. Additionally, MR-Egger intercept tests indicated potential horizontal pleiotropy for DVT, VTE, and PE. Our study suggests a potential role of erythritol in increasing the risk of coronary heart disease, ischemic stroke, and venous thromboembolism, which warrants further investigation.

## 1. Introduction

Erythritol, a widely used nonnutritive sugar alcohol, has gained increasing attention due to its rising presence in food products marketed for individuals with diabetes or those seeking healthier dietary alternatives.^[1,2]^ As a natural sweetener that provides negligible calories and does not elevate blood glucose or insulin levels, erythritol has long been considered metabolically benign. However, recent concerns have emerged regarding its potential impact on cardiovascular and thrombotic risk.^[3–6]^ Studies have shown that erythritol is endogenously produced in the human body via the pentose phosphate pathway and its circulating levels are closely associated with cardiometabolic phenotypes. Elevated erythritol concentrations have been reported in patients with obesity, diabetes, and cardiovascular diseases, suggesting its potential involvement in disease pathways beyond its role as a dietary additive.^[6,7]^

Mechanistic studies have further raised concerns about the biological effects of erythritol. In vivo and in vitro human trials suggest that erythritol may enhance platelet activation and increase the risk of thrombosis, providing biological plausibility for a link between erythritol and vascular events.^[8]^ Moreover, observational cohort studies have identified positive associations between higher plasma erythritol levels and major adverse cardiovascular events, including coronary heart disease (CHD) and stroke.^[2,9–11]^ However, observational studies were inherently limited in their ability to infer causality due to potential confounding and measurement error. Moreover, it remains unclear whether elevated erythritol levels are a cause or a consequence of underlying metabolic dysfunction.

To clarify the causal nature of these associations, Mendelian randomization (MR) provides a powerful tool by using genetic variants as instrumental variables to proxy long-term exposure to an exposure of interest—in this case, erythritol.^[12]^ By mimicking the randomization process of clinical trials, MR analyses can overcome many limitations of traditional observational studies, offering more robust evidence for causal inference. While prior MR analyses have attempted to evaluate the association between erythritol and certain cardiometabolic outcomes, the evidence remains limited, with some studies showing null associations and others lacking sufficient statistical power or methodological rigor.^[13]^ Moreover, no study to date has comprehensively evaluated the potential causal effects of erythritol on both arterial and venous thrombotic diseases in a unified framework.

In this study, we aimed to systematically assess the causal effect of genetically proxied erythritol on 5 major thrombotic events. To the best of our knowledge, this is among the first MR studies to investigate the potential role of erythritol in both arterial and venous thrombotic disorders. Our findings may provide novel insights into the vascular safety profile of erythritol and contribute to evidence-based recommendations for the use of nonnutritive sweeteners in clinical and public health settings.

## 2. Materials and methods

### 2.1. Study design

**Figure 1.**
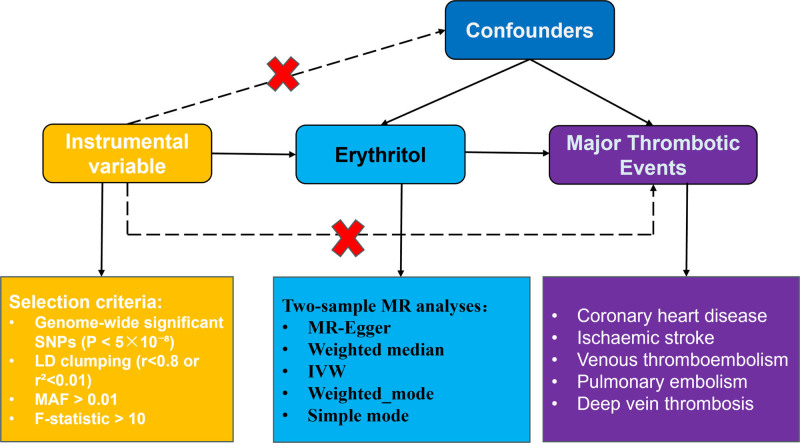
Study design overview. This study employed a 2-sample MR approach to investigate the potential causal effect of erythritol on major thrombotic events. The top panel illustrates the hypothesized causal pathways among genetic variants (SNPs), erythritol (exposure), and thrombotic events (outcomes). Solid arrows represent permitted causal relationships, while dashed lines indicate disallowed paths, which are essential for satisfying the instrumental variable assumptions. The bottom panel outlines the selection criteria for SNPs used as instrumental variables. Outcomes included coronary heart disease, ischemic stroke, venous thromboembolism (including deep vein thrombosis and pulmonary embolism), representing major thrombotic events. Summary-level data for both exposure and outcomes were obtained from large-scale GWAS. The primary MR method used was IVW, supplemented by multiple sensitivity analyses to assess robustness. GWAS = genome-wide association studies, IVW = inverse-variance weighted, LD = linkage disequilibrium, MR = Mendelian randomization, SNP = single nucleotide polymorphism.

Figure 1 illustrates the comprehensive design of our study. We conducted a 2-sample MR analysis to investigate the potential causal effects of erythritol on 5 major thrombotic events: coronary heart disease (CHD), ischemic stroke (IS), venous thromboembolism (VTE), pulmonary embolism (PE), and deep vein thrombosis (DVT). MR is an analytical framework that uses genetic variants as instrumental variables (IVs) to mimic the design of randomized controlled trials, thereby minimizing residual confounding.^[14,15]^ This approach relies on 3 key assumptions: the selected genetic variants must be strongly associated with the exposure (relevance), not associated with any confounders (independence), and must influence the outcome only through the exposure (exclusion restriction). This study used publicly available summary-level genome-wide association study (GWAS) data and did not involve individual-level participant data or ethical approval.

### 2.2. Data sources

Genetic instruments for erythritol were obtained from a GWAS including 8167 European individuals (GCST90199667), reported by Chen et al in 2023.^[16]^ Summary statistics for the outcomes were derived from the FinnGen consortium R10 release, also based on European ancestry populations (Table 1).

**Table 1:** Data sources.

Traits	Data source	Year	Sample size	Case	Control	Population	Data sources
Erythritol	Chen et al	2023	8167	NA	NA	European	https://www.ebi.ac.uk/gwas/studies/GCST90199667
Coronary heart disease	FinnGen	2023	412,181	46,959	365,222	European	https://storage.googleapis.com/finngen-public-data-r10/summary_stats/finngen_R10_I9_CHD.gz
Ischemic stroke	FinnGen	2023	399,220	27,497	371,723	European	https://storage.googleapis.com/finngen-public-data-r10/summary_stats/finngen_R10_I9_STR.gz
Venous thromboembolism	FinnGen	2023	412,181	21,021	391,160	European	https://storage.googleapis.com/finngen-public-data-r10/summary_stats/finngen_R10_I9_VTE.gz
Pulmonary embolism	FinnGen	2023	411,174	10,046	401,128	European	https://storage.googleapis.com/finngen-public-data-r10/summary_stats/finngen_R10_I9_PULMEMB.gz
Deep vein thrombosis	FinnGen	2023	363,612	6501	357,111	European	https://storage.googleapis.com/finngen-public-data-r10/summary_stats/finngen_R10_I9_PHLETHROMBDVTLOW.gz

### 2.3. Selection of instrumental variables

Single nucleotide polymorphisms (SNPs) strongly associated with erythritol (*P* < 5 × 10^‐8^) were selected. To ensure independence between instruments, linkage disequilibrium clumping was performed using a threshold of *R*^2^ < 0.01 within a 10,000 kb window. SNPs with a minor allele frequency < 0.01 or *F*-statistic < 10 were excluded to reduce weak instrument bias.^[17]^ *F*-statistic was calculated according to the formula *F* = ((n ‐ *k* ‐ 1)/*k*) * (*R*^2^/(1 ‐ *R*^2^)).^[17]^ n was the sample size of the exposed data, *k* represented the number of instruments and genetic variants (*R*^2^) was related to the proportion of variance in the exposure phenotype. These steps aimed to minimize weak instrument bias and improve the stability of the estimates. Palindromic SNPs with ambiguous strand orientation were removed.

### 2.4. Mendelian randomization analyses

The study findings were presented following the STROBE-MR (strengthening the reporting of Mendelian randomization studies) guidelines.^[18]^ The inverse-variance weighted (IVW) method was used as the primary MR analysis to estimate the causal effects of erythritol on major thrombotic events. The IVW method combines the ratio estimates of each SNP (i.e., SNP-outcome effect divided by SNP-exposure effect), weighted by their inverse variance.^[19]^ To account for potential horizontal pleiotropy, sensitivity analyses were performed using MR-Egger regression, weighted median, weighted mode, and simple mode approaches. MR-Egger intercept was used to test for directional pleiotropy,^[20]^ while Cochran *Q* statistic assessed heterogeneity across SNPs.^[15]^ To ensure the robustness of our findings, a result was deemed reliable only if it satisfied the following 3 criteria: the IVW method yielded a statistically significant *P*-value; there was no evidence of horizontal pleiotropy; and the effect directions estimated by the other MR methods—including MR-Egger regression, weighted median, weighted mode, and simple mode—were consistent with that of the IVW method. Additionally, to visually assess the influence of individual IVs on causal estimates and to evaluate the consistency and robustness of the results, we generated scatter plots, forest plots, leave-one-out sensitivity plots, and funnel plots.

Statistical analyses were performed using the “TwoSampleMR” R packages (Gibran Hemani at the MRC Integrative Epidemiology Unit, Bristol, United Kingdom). *P* < .05/5 (with Bonferroni corrections) was statistically significant with *P*-value between .05 and .01 as suggestively significant.

## 3. Results

### 3.1. IV selection

We selected 60 independent SNPs strongly associated with erythritol (*P* < 5 × 10^‐8^), with *F* statistics ranging from 25.79 to 94.13, indicating no weak instrument bias. Palindromic variants were removed (Table S1, Supplemental Digital Content, https://links.lww.com/MD/Q430). These IVs collectively explained a sufficient proportion of variance in erythritol levels and satisfied MR assumptions.

### 3.2. Effect of genetically proxied erythritol on 5 major thrombotic events

**Figure 2.**
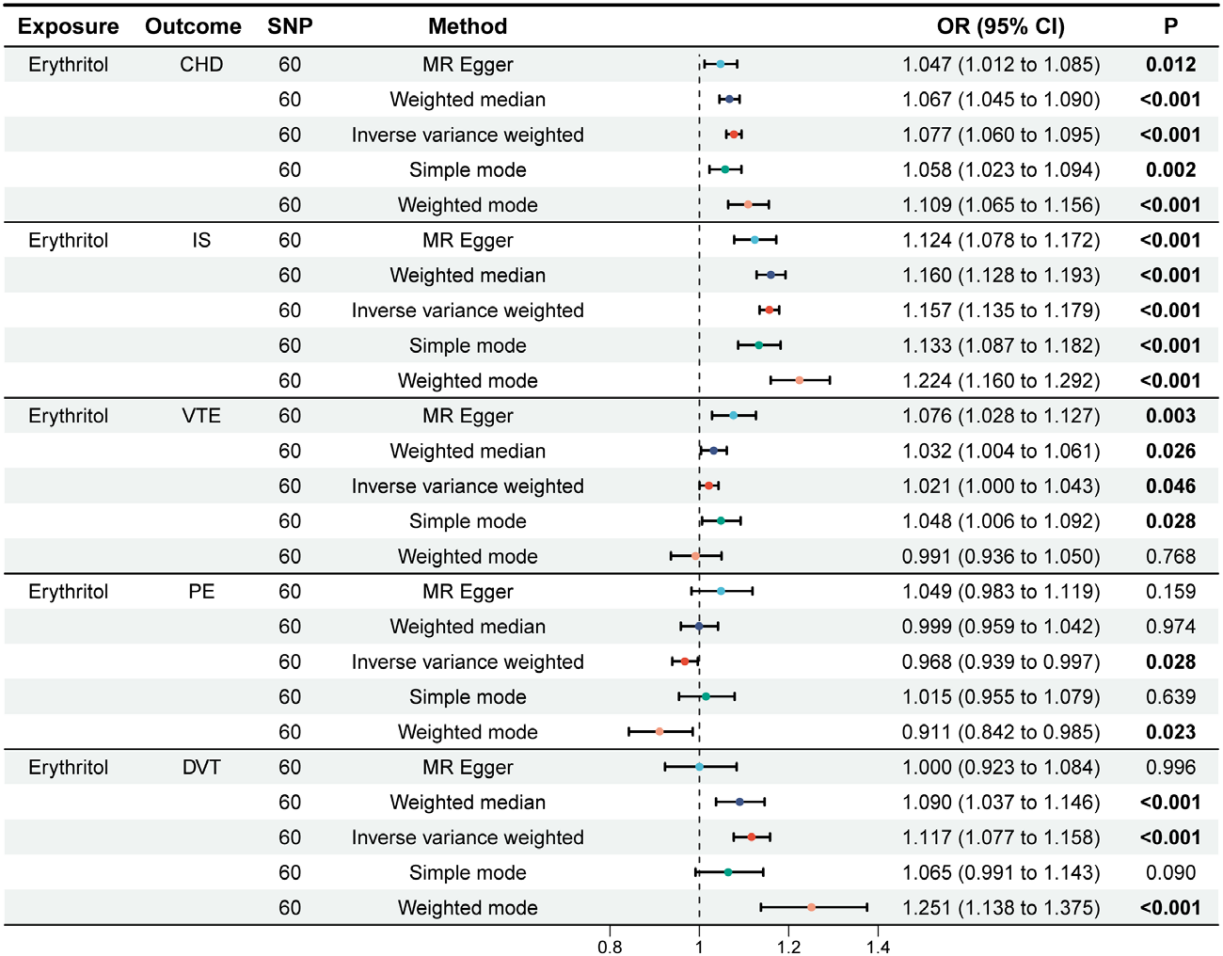
The causal effect of erythritol on 5 major thrombotic events. CHD = coronary heart disease, DVT = deep vein thrombosis, IS = ischemic stroke, PE = pulmonary embolism, SNP = single nucleotide polymorphism, VTE = venous thromboembolism.

The IVW method showed significant associations of genetically predicted erythritol with increased risks of CHD (odds ratio [OR] = 1.077, 95% confidence interval [CI]: 1.060–1.090, *P* < .001), IS (OR = 1.157, 95% CI: 1.135–1.179, *P* < .001), and DVT (OR = 1.117, 95% CI: 1.077–1.158, *P* < .001). Suggestive associations were found for VTE (OR = 1.021, 95% CI: 1.000–1.043, *P* = .046) and PE (OR = 0.968, 95% CI: 0.939–0.997, *P* = .028). Other MR methods (MR-Egger, weighted median, weighted mode, and simple mode) yielded similar or partially consistent results (Fig. 2).

### 3.3. Sensitivity analyses

**Figure 3.**
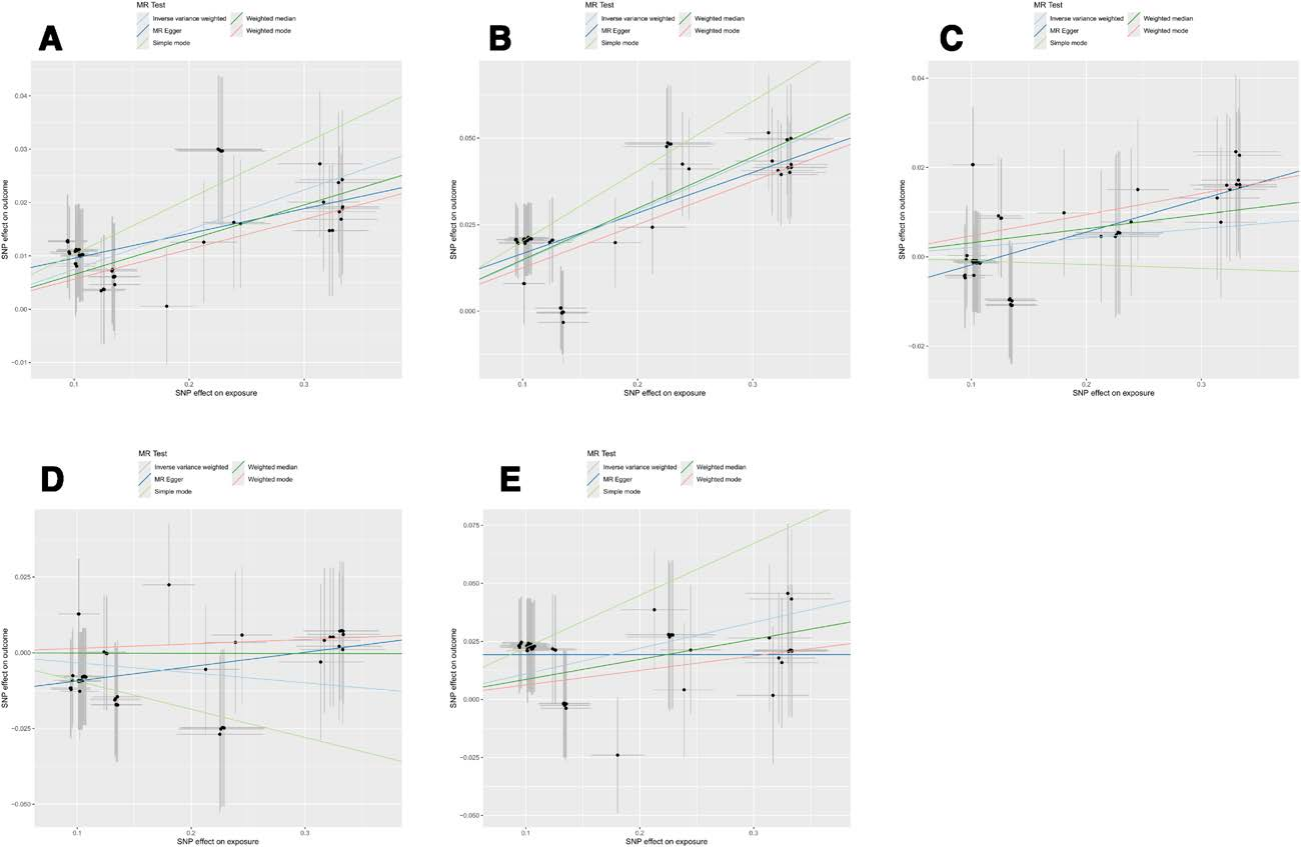
The scatter plots for causal effect of erythritol on 5 major thrombotic events. (A) Erythritol on coronary heart disease, (B) erythritol on ischemic stroke, (C) erythritol on venous thromboembolism, (D) erythritol on pulmonary embolism, and (E) erythritol on deep vein thrombosis.

Sensitivity analyses showed no evidence of directional pleiotropy for CHD and IS (MR-Egger intercept *P* > .05). Cochran *Q* test did not indicate significant heterogeneity. Leave-one-out analysis showed that causal estimates were not driven by any single SNP (Figure S1, Supplemental Digital Content, https://links.lww.com/MD/Q429). Funnel plots were symmetric for most outcomes (Figure S2, Supplemental Digital Content, https://links.lww.com/MD/Q429). Scatter plots and forest plots were presented in Figure 3 and Figure S3, Supplemental Digital Content, https://links.lww.com/MD/Q429.

In contrast, for VTE and PE, the causal effect directions estimated by the other 4 MR methods were not consistent with that of the IVW method. Moreover, the MR-Egger intercept indicated potential horizontal pleiotropy in VTE, PE, and DVT (Table S2, Supplemental Digital Content, https://links.lww.com/MD/Q430). Together, these findings suggest that although the IVW analysis supported an association, the evidence of pleiotropy from alternative MR methods indicates that part of the genetic effect may act through undefined pathways beyond erythritol.

## 4. Discussion

In this 2-sample MR study, we found genetic evidence supporting a potential causal effect of erythritol on CHD and IS. These associations remained robust across multiple MR methods and sensitivity analyses, showing consistent effect directions, no significant heterogeneity, and no evidence of directional pleiotropy. In contrast, we found no reliable evidence to support a causal relationship between erythritol and venous thromboembolic diseases, including DVT, PE, and VTE. These findings suggest that erythritol may specifically influence arterial rather than venous thrombotic pathways.

Our research findings are highly consistent with existing mechanistic studies, suggesting that erythritol increases the risk of arterial thrombotic events such as CHD and IS through multiple biological pathways. Previous metabolomics studies have found that elevated circulating erythritol levels are significantly associated with the incidence risk of myocardial infarction and stroke within 3 years, and this association is consistent across different genders, ethnicities, and regions (including populations in the United States and Europe), independent of traditional cardiovascular metabolic risk factors.^[8]^ To further validate its biological mechanisms, prospective intervention trials have shown that intake of 30 grams of erythritol (equivalent to the amount in a typical can of sugar-free beverage) significantly enhances platelet activity and increases thrombogenic potential; this effect has been clearly demonstrated in both in vitro flow models and animal arterial injury models.^[1,21]^ In addition, other studies have reported an independent positive association between higher circulating erythritol levels and increased risks of cardiovascular disease and all-cause mortality.^[9,22,23]^ Additionally, erythritol has been shown to increase oxidative stress, reduce nitric oxide bioavailability, and promote endothelin-1 (ET-1) production, thereby impairing the fibrinolytic capacity of brain microvascular endothelial cells in vitro.^[24]^ These pathological changes—namely increased oxidative stress, decreased NO production, enhanced ET-1 system activity, and impaired fibrinolysis—are core features of cerebral endothelial dysfunction and key contributors to the development, severity, and outcome of ischemic stroke.^[25]^ This evidence supports and experimentally extends epidemiological findings linking erythritol to an elevated risk of stroke. Collectively, these findings suggest that erythritol may promote arterial thrombosis and cerebrovascular dysfunction by enhancing platelet adhesion and activation, accelerating atherosclerosis, increasing oxidative stress, reducing nitric oxide bioavailability, and elevating ET-1 production, thereby contributing to the increased risk of CHD and IS.

Nevertheless, evidence from observational studies is susceptible to residual confounding and measurement bias. Specifically, elevated erythritol levels may not be a causal factor but rather a metabolic consequence of cardiometabolic diseases such as obesity, insulin resistance, or type 2 diabetes. To overcome these limitations, our study applied MR, which leverages genetic variants as instrumental variables to mimic lifelong erythritol exposure. This approach reduces confounding and reverse causation, allowing for a more reliable assessment of causality. The consistency of our MR results with mechanistic evidence suggests a potential association between erythritol and the risk of CHD and IS, but causal inference should be interpreted with caution.

Interestingly, our findings did not support a causal effect of erythritol on VTE, PE, and DVT. Although both venous and arterial thrombosis involve pathological blood clot formation, they differ fundamentally in pathophysiological mechanisms. DVT is primarily driven by venous stasis, hypercoagulability, and endothelial injury—components of Virchow triad—and typically occurs under low-shear conditions.^[26,27]^ In contrast, arterial thrombosis often results from endothelial dysfunction, platelet activation, and atherosclerotic plaque rupture, processes that take place in high-shear arterial environments and are central to the pathogenesis of cardiovascular events such as CHD and IS.^[28–30]^ Importantly, the known biological effects of erythritol—such as increasing oxidative stress, reducing NO bioavailability, enhancing ET-1 production, and impairing fibrinolytic capacity in brain microvascular endothelial cells—are key contributors to arterial endothelial dysfunction rather than venous stasis. Furthermore, erythritol has been shown to promote platelet adhesion and activation, which are critical in arterial thrombus formation but less central in DVT, where activation of the coagulation cascade predominates. Additionally, erythritol may accelerate atherosclerotic processes, further supporting its potential role in arterial rather than VTE. Consistent with these mechanistic differences, MR analyses for VTE, PE, and DVT showed inconsistent causal directions and evidence of horizontal pleiotropy—such as nonzero MR-Egger intercepts and divergent mode-based estimates—suggesting that the observed associations may be confounded or noncausal. Therefore, the evidence for a causal effect of erythritol on VTE is weak and should be interpreted with caution.

Our study has several strengths. It is the first MR study to systematically evaluate both arterial and venous thrombotic events in relation to genetically predicted erythritol levels. We used a comprehensive set of SNPs from a recent GWAS and applied multiple complementary MR methods to test the robustness of our findings. The use of large-scale, high-quality outcome data from the FinnGen consortium provided sufficient power to detect modest effect sizes. However, some limitations should be acknowledged. First, our study population consisted of Europeans only, which limits the generalizability of the findings to other ethnic groups. Second, due to multiple testing, some findings may represent potential false positives. Third, while our genetic instruments reflect long-term endogenous erythritol exposure, they may not fully capture the acute effects of dietary intake, which could introduce discrepancies between genetic proxies and actual exposure. Fourth, although MR helps reduce confounding and reverse causation, it depends on key assumptions—such as the absence of horizontal pleiotropy and the validity of the instrumental variables—which, if violated, could bias the estimates. Further mechanistic and clinical studies across diverse populations are needed to validate and extend our findings.

In summary, our MR study provides evidence for a potential association between erythritol and an increased risk of arterial thrombotic events, including CHD and IS. However, we did not find robust evidence supporting a causal effect on venous thrombotic events. These findings highlight the importance of further mechanistic and clinical studies to evaluate erythritol’s vascular safety, particularly in high-risk populations. Overall, while the results suggest a potential link, causal interpretations should be made with caution, and additional research is warranted to confirm these observations.

## Author contributions

**Conceptualization:** Juan Jin.

**Data curation:** Yao Sun.

**Formal analysis:** Ze Peng.

**Funding acquisition:** Juan Jin.

**Investigation:** Juan Jin.

**Methodology:** Jinqiao Wu.

**Project administration:** Juan Jin.

**Resources:** Dingxin Sun, Ze Peng.

**Software:** Dingxin Sun, Jinqiao Wu.

**Supervision:** Jinqiao Wu, Ze Peng.

**Validation:** Yao Sun.

**Visualization:** Yao Sun.

**Writing – original draft:** Yao Sun, Dingxin Sun, Ze Peng, Juan Jin.

**Writing – review & editing:** Yao Sun, Dingxin Sun, Jinqiao Wu.

## Supplementary Material


