# Supplementary material for: Role of erythritol in coronary heart disease, ischemic stroke, and venous thromboembolism: A Mendelian randomization analysis
Source: Medicine (Baltimore). 2025 Oct 24;104(43):e45187. doi: 10.1097/MD.0000000000045187 (PMC12558269; doi:10.1097/MD.0000000000045187)
Supplement: Supplementary file 1 [file medi-104-e45187-s001.docx]

**Supplementary Table 1.** Characteristics of SNPs associated with erythritol.

| **SNP** | **Effect allele/**  **Non-effect allele** | **Effect allele frequency** | **Beta** | **Se** | **P values** | **N** | **R^2^** | **F statistics** | **Palindromic** |
| --- | --- | --- | --- | --- | --- | --- | --- | --- | --- |
| rs1053941 | T/G | 0.235 | -0.105 | 0.017 | 4.67E-10 | 8167 | 3.96E-03 | 32.50 | FALSE |
| rs11211133 | A/G | 0.232 | -0.095 | 0.017 | 2.81E-08 | 8167 | 3.22E-03 | 26.34 | FALSE |
| rs112260114 | A/G | 0.241 | -0.103 | 0.017 | 7.06E-10 | 8167 | 3.88E-03 | 31.81 | FALSE |
| rs11579176 | T/G | 0.242 | -0.102 | 0.017 | 9.34E-10 | 8167 | 3.82E-03 | 31.28 | FALSE |
| rs11585275 | C/T | 0.235 | -0.106 | 0.017 | 3.13E-10 | 8167 | 4.04E-03 | 33.12 | FALSE |
| rs11587982 | T/A | 0.241 | -0.103 | 0.017 | 9.04E-10 | 8167 | 3.88E-03 | 31.81 | TRUE |
| rs11590549 | T/C | 0.244 | -0.102 | 0.017 | 7.95E-10 | 8167 | 3.84E-03 | 31.46 | FALSE |
| rs12021717 | A/G | 0.232 | -0.094 | 0.017 | 3.40E-08 | 8167 | 3.15E-03 | 25.79 | FALSE |
| rs12042481 | C/T | 0.162 | -0.124 | 0.020 | 2.46E-10 | 8167 | 4.17E-03 | 34.23 | FALSE |
| rs12047498 | T/C | 0.232 | -0.094 | 0.017 | 2.94E-08 | 8167 | 3.15E-03 | 25.79 | FALSE |
| rs140872745 | C/A | 0.038 | -0.226 | 0.038 | 2.09E-09 | 8167 | 3.73E-03 | 30.60 | FALSE |
| rs2065708 | G/A | 0.232 | -0.094 | 0.017 | 3.40E-08 | 8167 | 3.15E-03 | 25.79 | FALSE |
| rs2229540 | G/A | 0.054 | -0.322 | 0.032 | 4.20E-24 | 8167 | 1.06E-02 | 87.42 | FALSE |
| rs28412451 | G/A | 0.241 | -0.104 | 0.017 | 5.66E-10 | 8167 | 3.96E-03 | 32.44 | FALSE |
| rs28433203 | T/G | 0.241 | -0.104 | 0.017 | 5.96E-10 | 8167 | 3.96E-03 | 32.44 | FALSE |
| rs28484896 | G/C | 0.241 | -0.104 | 0.017 | 4.54E-10 | 8167 | 3.96E-03 | 32.44 | TRUE |
| rs28584711 | T/C | 0.234 | -0.108 | 0.017 | 1.85E-10 | 8167 | 4.18E-03 | 34.28 | FALSE |
| rs28733899 | G/C | 0.234 | -0.107 | 0.017 | 2.25E-10 | 8167 | 4.10E-03 | 33.65 | TRUE |
| rs3014232 | C/T | 0.243 | -0.101 | 0.017 | 1.28E-09 | 8167 | 3.75E-03 | 30.76 | FALSE |
| rs41310432 | G/C | 0.236 | -0.105 | 0.017 | 3.83E-10 | 8167 | 3.98E-03 | 32.59 | TRUE |
| rs4687718 | G/A | 0.875 | 0.133 | 0.022 | 9.79E-10 | 8167 | 3.87E-03 | 31.72 | FALSE |
| rs55659201 | A/G | 0.241 | -0.104 | 0.017 | 5.90E-10 | 8167 | 3.96E-03 | 32.44 | FALSE |
| rs55951835 | C/T | 0.243 | -0.101 | 0.017 | 1.39E-09 | 8167 | 3.75E-03 | 30.76 | FALSE |
| rs56020747 | T/C | 0.242 | -0.101 | 0.017 | 1.16E-09 | 8167 | 3.74E-03 | 30.67 | FALSE |
| rs56067046 | T/C | 0.235 | -0.105 | 0.017 | 4.61E-10 | 8167 | 3.96E-03 | 32.50 | FALSE |
| rs56093189 | C/T | 0.244 | -0.096 | 0.017 | 9.96E-09 | 8167 | 3.40E-03 | 27.86 | FALSE |
| rs56108122 | T/G | 0.054 | -0.333 | 0.032 | 1.63E-25 | 8167 | 1.13E-02 | 93.56 | FALSE |
| rs56174961 | T/A | 0.040 | -0.302 | 0.037 | 2.55E-16 | 8167 | 7.00E-03 | 57.59 | TRUE |
| rs56238114 | G/A | 0.242 | -0.101 | 0.017 | 1.18E-09 | 8167 | 3.74E-03 | 30.67 | FALSE |
| rs56251392 | T/C | 0.234 | -0.107 | 0.017 | 2.20E-10 | 8167 | 4.10E-03 | 33.65 | FALSE |
| rs56261438 | T/C | 0.166 | -0.126 | 0.019 | 8.39E-11 | 8167 | 4.40E-03 | 36.05 | FALSE |
| rs58692133 | A/G | 0.242 | -0.102 | 0.017 | 1.01E-09 | 8167 | 3.82E-03 | 31.28 | FALSE |
| rs62255992 | C/A | 0.123 | -0.135 | 0.022 | 8.35E-10 | 8167 | 3.93E-03 | 32.23 | FALSE |
| rs62255995 | G/A | 0.123 | -0.134 | 0.022 | 1.04E-09 | 8167 | 3.87E-03 | 31.75 | FALSE |
| rs62255996 | G/A | 0.123 | -0.134 | 0.022 | 1.04E-09 | 8167 | 3.87E-03 | 31.75 | FALSE |
| rs62256001 | G/A | 0.121 | -0.135 | 0.022 | 9.10E-10 | 8167 | 3.88E-03 | 31.78 | FALSE |
| rs62256002 | C/T | 0.122 | -0.133 | 0.022 | 1.48E-09 | 8167 | 3.79E-03 | 31.06 | FALSE |
| rs6429573 | A/G | 0.243 | -0.103 | 0.017 | 6.39E-10 | 8167 | 3.90E-03 | 31.99 | FALSE |
| rs6662572 | G/A | 0.242 | -0.104 | 0.017 | 4.64E-10 | 8167 | 3.97E-03 | 32.53 | FALSE |
| rs6662999 | C/G | 0.235 | -0.106 | 0.017 | 3.16E-10 | 8167 | 4.04E-03 | 33.12 | TRUE |
| rs6671239 | C/A | 0.236 | -0.106 | 0.017 | 2.52E-10 | 8167 | 4.05E-03 | 33.22 | FALSE |
| rs6688727 | G/A | 0.040 | -0.314 | 0.037 | 1.58E-17 | 8167 | 7.57E-03 | 62.30 | FALSE |
| rs72676583 | A/T | 0.037 | -0.225 | 0.038 | 2.99E-09 | 8167 | 3.61E-03 | 29.56 | TRUE |
| rs72676586 | T/G | 0.037 | -0.225 | 0.038 | 2.99E-09 | 8167 | 3.61E-03 | 29.56 | FALSE |
| rs72676587 | G/A | 0.037 | -0.229 | 0.038 | 1.54E-09 | 8167 | 3.74E-03 | 30.63 | FALSE |
| rs72676591 | C/T | 0.037 | -0.228 | 0.038 | 1.85E-09 | 8167 | 3.70E-03 | 30.36 | FALSE |
| rs72677528 | G/A | 0.053 | -0.331 | 0.032 | 5.94E-25 | 8167 | 1.10E-02 | 90.80 | FALSE |
| rs72677556 | C/A | 0.199 | -0.101 | 0.018 | 1.80E-08 | 8167 | 3.25E-03 | 26.64 | FALSE |
| rs72677557 | C/T | 0.199 | -0.101 | 0.018 | 2.03E-08 | 8167 | 3.25E-03 | 26.64 | FALSE |
| rs72677559 | G/C | 0.199 | -0.101 | 0.018 | 1.80E-08 | 8167 | 3.25E-03 | 26.64 | TRUE |
| rs72677566 | A/G | 0.065 | -0.213 | 0.029 | 3.62E-13 | 8167 | 5.51E-03 | 45.28 | FALSE |
| rs72686459 | T/C | 0.109 | -0.181 | 0.023 | 6.26E-15 | 8167 | 6.36E-03 | 52.29 | FALSE |
| rs72686491 | T/G | 0.054 | -0.317 | 0.032 | 1.60E-23 | 8167 | 1.03E-02 | 84.70 | FALSE |
| rs72686493 | G/A | 0.077 | -0.239 | 0.027 | 6.60E-19 | 8167 | 8.12E-03 | 66.84 | FALSE |
| rs72688441 | A/G | 0.054 | -0.325 | 0.032 | 1.66E-24 | 8167 | 1.08E-02 | 89.07 | FALSE |
| rs72688452 | A/G | 0.243 | -0.102 | 0.017 | 9.85E-10 | 8167 | 3.83E-03 | 31.37 | FALSE |
| rs72688460 | T/C | 0.235 | -0.106 | 0.017 | 3.10E-10 | 8167 | 4.04E-03 | 33.12 | FALSE |
| rs72690839 | T/G | 0.053 | -0.332 | 0.032 | 3.12E-25 | 8167 | 1.11E-02 | 91.35 | FALSE |
| rs72690858 | G/A | 0.039 | -0.330 | 0.038 | 1.77E-18 | 8167 | 8.16E-03 | 67.20 | FALSE |
| rs72692616 | A/G | 0.054 | -0.334 | 0.032 | 1.56E-25 | 8167 | 1.14E-02 | 94.13 | FALSE |
| rs72692619 | G/T | 0.039 | -0.333 | 0.037 | 3.65E-19 | 8167 | 8.31E-03 | 68.44 | FALSE |
| rs72692627 | C/T | 0.077 | -0.245 | 0.027 | 6.47E-20 | 8167 | 8.53E-03 | 70.26 | FALSE |
| rs73840299 | T/C | 0.123 | -0.135 | 0.022 | 8.35E-10 | 8167 | 3.93E-03 | 32.23 | FALSE |
| rs7517111 | G/A | 0.243 | -0.103 | 0.017 | 6.95E-10 | 8167 | 3.90E-03 | 31.99 | FALSE |
| rs7520156 | T/A | 0.055 | -0.318 | 0.032 | 7.60E-24 | 8167 | 1.05E-02 | 86.74 | TRUE |
| rs7541935 | T/C | 0.241 | -0.104 | 0.017 | 4.51E-10 | 8167 | 3.96E-03 | 32.44 | FALSE |
| rs7553374 | A/G | 0.166 | -0.126 | 0.019 | 7.00E-11 | 8167 | 4.40E-03 | 36.05 | FALSE |
| rs76785074 | C/G | 0.113 | -0.132 | 0.023 | 7.21E-09 | 8167 | 3.49E-03 | 28.62 | TRUE |
| rs77351539 | C/G | 0.123 | -0.134 | 0.022 | 1.04E-09 | 8167 | 3.87E-03 | 31.75 | TRUE |
| rs9429173 | C/A | 0.251 | -0.096 | 0.017 | 7.13E-09 | 8167 | 3.47E-03 | 28.39 | FALSE |
| rs9858049 | T/C | 0.123 | -0.135 | 0.022 | 6.76E-10 | 8167 | 3.93E-03 | 32.23 | FALSE |

SNP, single-nucleotide polymorphism; The F-statistic for each SNP was calculated as follows: F =((N-2)*(R^2^/ (1− R^2^)), R² was calculated as follows: 2*BETA^2*EAF*(1-EAF).

**Supplementary Table 2.** Two-sample MR analyses of genetically proxied erythritol on major thrombotic events.

| **Exposure** | **Outcomes** | **Methods** | **N snp** | **β（95% CI)** | **P values** | **P-het** | **P-ple** |
| --- | --- | --- | --- | --- | --- | --- | --- |
| Erythritol | Coronary heart disease | MR Egger | 60 | 0.05(0.01,0.08) | 0.012 | 1 | 0.081 |
|  |  | Weighted median | 60 | 0.07(0.04,0.09) | <0.001 | 1 |  |
|  |  | Inverse variance weighted | 60 | 0.07(0.06,0.09) | <0.001 |  |  |
|  |  | Weighted mode | 60 | 0.06(0.02,0.09) | 0.002 |  |  |
|  |  | Simple mode | 60 | 0.10(0.06,0.14) | <0.001 |  |  |
| Erythritol | Ischaemic stroke | MR Egger | 60 | 0.12(0.08,0.16) | <0.001 | 0.991 | 0.140 |
|  |  | Weighted median | 60 | 0.15(0.12,0.18) | <0.001 | 0.986 |  |
|  |  | Inverse variance weighted | 60 | 0.15(0.13,0.16) | <0.001 |  |  |
|  |  | Weighted mode | 60 | 0.13(0.08,0.17) | <0.001 |  |  |
|  |  | Simple mode | 60 | 0.20(0.15,0.26) | <0.001 |  |  |
| Erythritol | Venous thromboembolism | MR Egger | 60 | 0.07(0.03,0.12) | 0.003 | 1 | 0.014 |
|  |  | Weighted median | 60 | 0.03(0,0.06) | 0.026 | 1 |  |
|  |  | Inverse variance weighted | 60 | 0.02(0,0.04) | 0.046 |  |  |
|  |  | Weighted mode | 60 | 0.05(0.01,0.09) | 0.028 |  |  |
|  |  | Simple mode | 60 | -0.01(-0.07,0.05) | 0.768 |  |  |
| Erythritol | Pulmonary embolism | MR Egger | 60 | 0.05(-0.02,0.11) | 0.159 | 1 | 0.009 |
|  |  | Weighted median | 60 | 0(-0.04,0.04) | 0.974 | 1 |  |
|  |  | Inverse variance weighted | 60 | -0.03(-0.06,0) | 0.028 |  |  |
|  |  | Weighted mode | 60 | 0.01(-0.05,0.08) | 0.639 |  |  |
|  |  | Simple mode | 60 | -0.09(-0.17,-0.01) | 0.023 |  |  |
| Erythritol | Deep vein thrombosis | MR Egger | 60 | 0(-0.08,0.08) | 0.996 | 1 | 0.004 |
|  |  | Weighted median | 60 | 0.09(0.04,0.14) | 0.001 | 1 |  |
|  |  | Inverse variance weighted | 60 | 0.11(0.07,0.15) | <0.001 |  |  |
|  |  | Weighted mode | 60 | 0.06(-0.01,0.13) | 0.090 |  |  |
|  |  | Simple mode | 60 | 0.22(0.13,0.32) | <0.001 |  |  |

SNP, single nucleotide polymorphism; IVW, inverse variance weighted; CI, confidence interval; P-het, p value for heterogeneity test; P-ple, p value for pleiotropy test.
