# Supplementary material for: Role of erythritol in coronary heart disease, ischemic stroke, and venous thromboembolism: A Mendelian randomization analysis
Source: Medicine (Baltimore). 2025 Oct 24;104(43):e45187. doi: 10.1097/MD.0000000000045187 (PMC12558269; doi:10.1097/MD.0000000000045187)
Supplement: Supplementary file 2 [file medi-104-e45187-s002.docx]

**Supplementary Figure 1.** The leave-one-out analysis for causal effect of erythritol on five major thrombotic events.


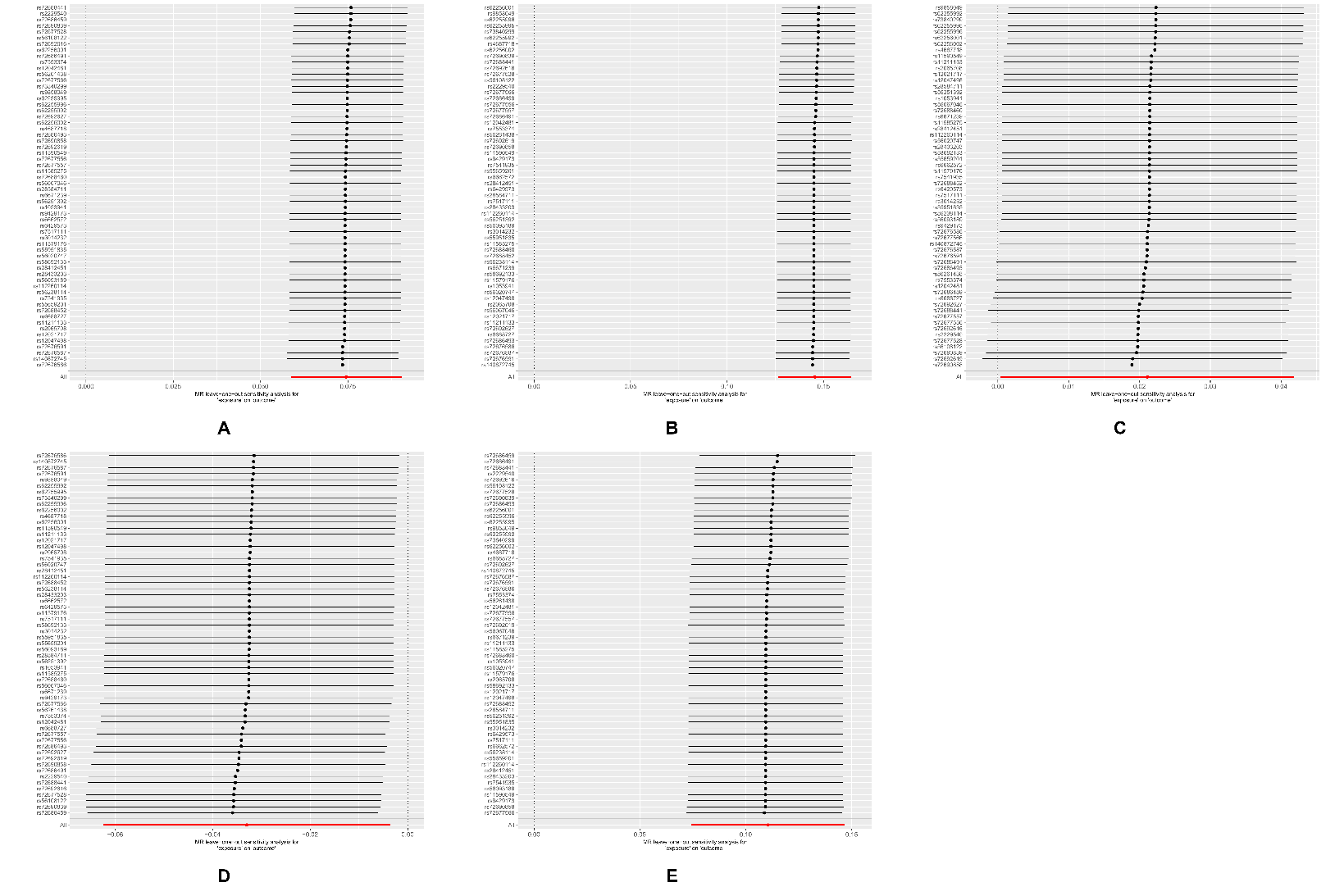


(A) Erythritol on coronary heart disease, (B) Erythritol on ischaemic stroke, (C) Erythritol on venous thromboembolism, (D) Erythritol on pulmonary embolism, (E) Erythritol on deep vein thrombosis.

**Supplementary Figure 2.** The funnel plots for causal effect of erythritol on five major thrombotic events.


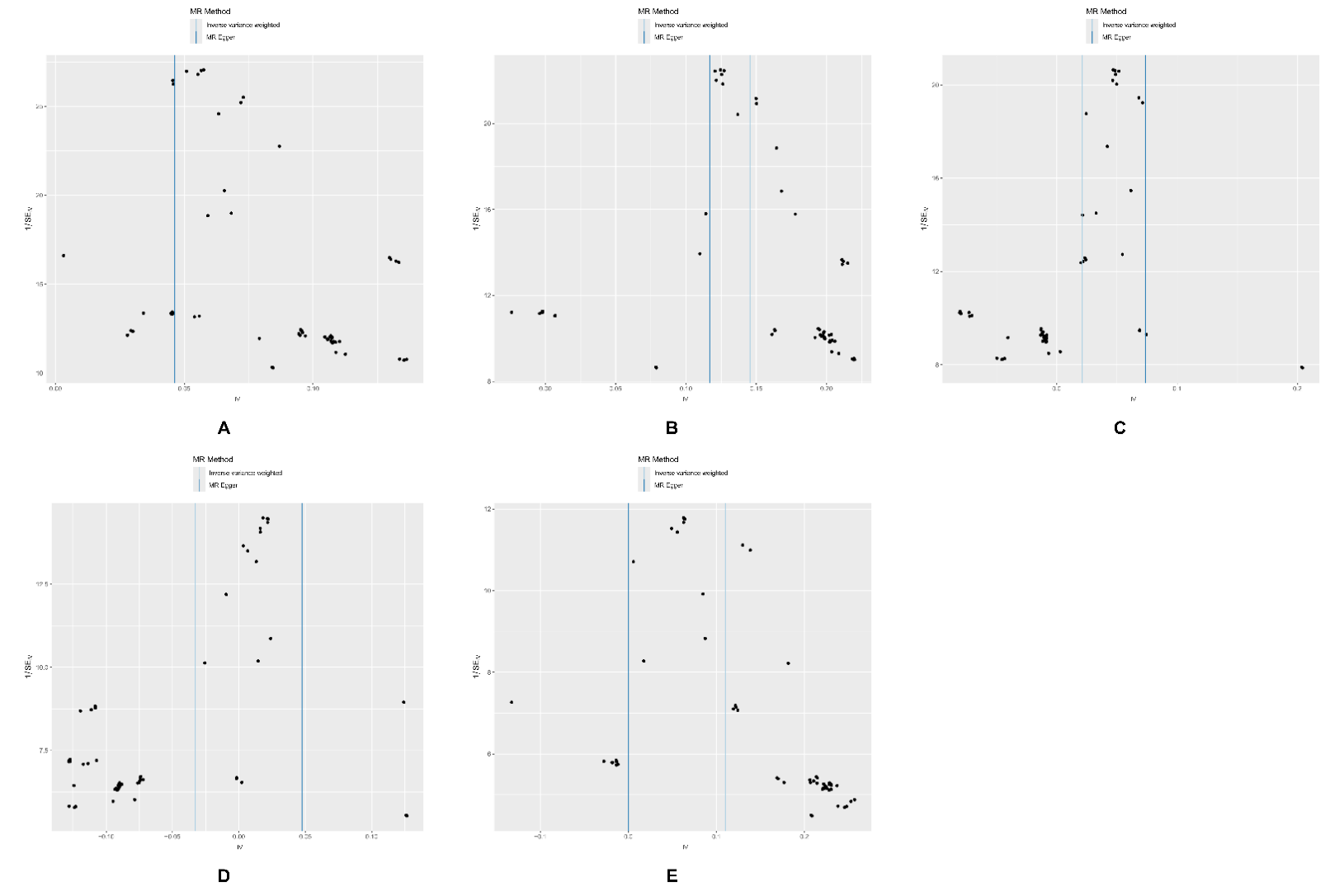


(A) Erythritol on coronary heart disease, (B) Erythritol on ischaemic stroke, (C) Erythritol on venous thromboembolism, (D) Erythritol on pulmonary embolism, (E) Erythritol on deep vein thrombosis.

**Supplementary Figure 3.** The forest plots for causal effect of erythritol on five major thrombotic events.


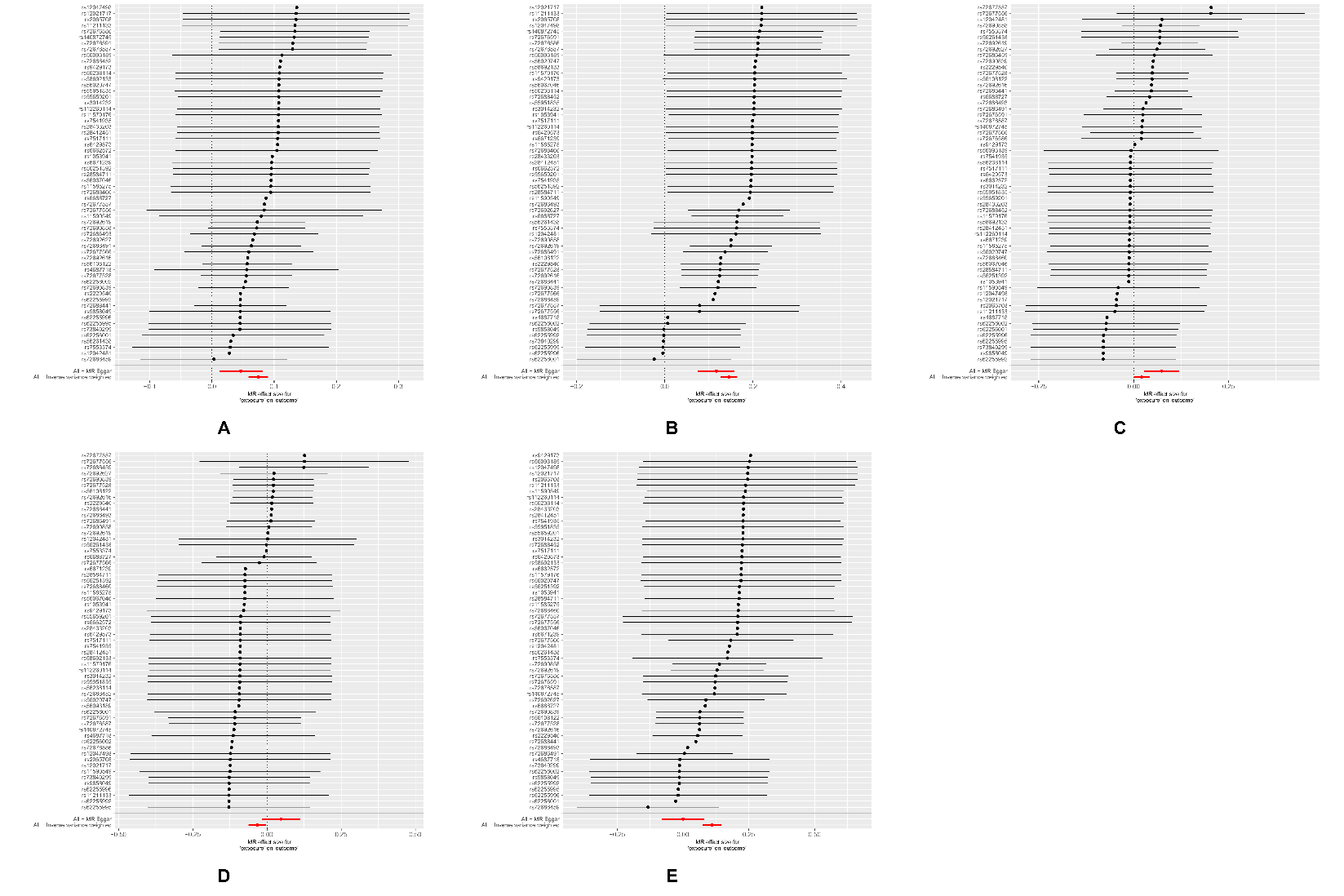


(A) Erythritol on coronary heart disease, (B) Erythritol on ischaemic stroke, (C) Erythritol on venous thromboembolism, (D) Erythritol on pulmonary embolism, (E) Erythritol on deep vein thrombosis.
